# Poly[aqua­(μ_11_-4,6-dihy­droxy­benzene-1,3-disulfonato)­dipotassium]

**DOI:** 10.1107/S1600536811047210

**Published:** 2011-11-12

**Authors:** Zhu-Lin Xie, Wu-Leng Lai, Rui-Qing Yang, Yong-Rong Xie

**Affiliations:** a School of Chemistry and Chemical Engineering, University of Jinan, Jinan 250022, People’s Republic of China; b Key Laboratory of Jiangxi University for Functional Materials Chemistry, College of Chemistry and Chemical Engineering, Gannan Normal University, Ganzhou 341000, People’s Republic of China

## Abstract

In the title salt, [K_2_(C_6_H_4_O_8_S_2_)(H_2_O)]_*n*_, both K^+^ ions exhibit a seven-coordination with K—O bond lengths in the range 2.6600 (14) to 3.0522 (16) Å. One K^+^ ion is coordinated by seven O atoms from the sulfonate and phenolic hy­droxy groups of six 4,6-dihy­droxy­benzene-1,3-disulfonate (*L* ^2−^) anions while the other K^+^ ion is coordinated by six O atoms from the sulfonate and phenolic hy­droxy groups of five *L* ^2−^ anions and one water O atom. The *L* ^2−^ anion exhibits chelating–bridging multidentate coordination to potassium, resulting in the formation of a cross-linked three-dimensional network.

## Related literature

For K—O bond lengths of potassium complexes, see: Hatano *et al.* (2008 ▶); Xie *et al.* (2006 ▶); Zhang *et al.* (2006 ▶). For other complexes with seven-coordinate potassium atoms, see: Deacon *et al.* (1999 ▶).
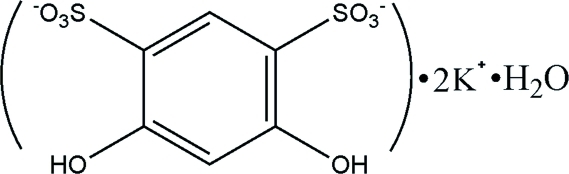


## Experimental

### 

#### Crystal data

[K_2_(C_6_H_4_O_8_S_2_)(H_2_O)]
*M* *_r_* = 364.43
Monoclinic, 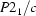

*a* = 7.3427 (3) Å
*b* = 11.5194 (5) Å
*c* = 14.6331 (6) Å
β = 100.495 (1)°
*V* = 1217.01 (9) Å^3^
*Z* = 4
Mo *K*α radiation
μ = 1.16 mm^−1^
*T* = 292 K
0.33 × 0.20 × 0.17 mm

#### Data collection

Bruker SMART APEXII CCD area-detector diffractometer
Absorption correction: multi-scan (*SADABS*; Sheldrick, 1996 ▶) *T*_min_ = 0.753, *T*_max_ = 0.816
6519 measured reflections
2365 independent reflections
2164 reflections with *I* > 2σ(*I*)
*R*_int_ = 0.013

#### Refinement

*R*[*F* ^2^ > 2σ(*F* ^2^)] = 0.024
*wR*(*F* ^2^) = 0.071
*S* = 1.08
2365 reflections
173 parameters
H-atom parameters constrained
Δρ_max_ = 0.38 e Å^−3^
Δρ_min_ = −0.33 e Å^−3^

### 

Data collection: *APEX2* (Bruker, 2007 ▶); cell refinement: *SAINT* (Bruker, 2007 ▶); data reduction: *SAINT*; program(s) used to solve structure: *SHELXS97* (Sheldrick, 2008 ▶); program(s) used to refine structure: *SHELXL97* (Sheldrick, 2008 ▶); molecular graphics: *SHELXTL* (Sheldrick, 2008 ▶); software used to prepare material for publication: *SHELXTL*.

## Supplementary Material

Crystal structure: contains datablock(s) I, global. DOI: 10.1107/S1600536811047210/vm2127sup1.cif

Structure factors: contains datablock(s) I. DOI: 10.1107/S1600536811047210/vm2127Isup2.hkl

Additional supplementary materials:  crystallographic information; 3D view; checkCIF report

## Figures and Tables

**Table 1:** Hydrogen-bond geometry (Å, °)

*D*—H⋯*A*	*D*—H	H⋯*A*	*D*⋯*A*	*D*—H⋯*A*
O8—H8*A*⋯O4	0.82	1.88	2.625 (2)	151
O7—H7*A*⋯O1	0.82	1.86	2.613 (2)	152
O9—H9*A*⋯O6^i^	0.85	2.29	2.916 (2)	130
O9—H9*B*⋯O2^ii^	0.85	2.26	2.913 (3)	134

Symmetry codes: (i) 
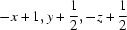
; (ii) 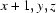.

**Fig. 1.**
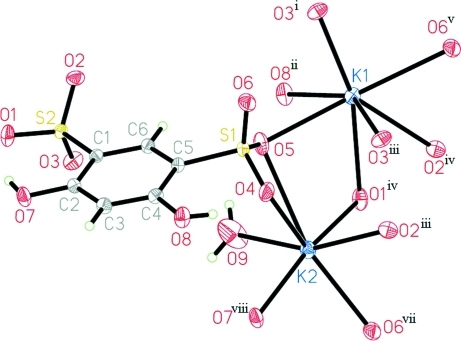
The coordination environment of the K+ ions showing displacement ellipsoids drawn at the 30% probability level. [Symmetry codes: (i) x, -y + 1/2, z + 1/2; (ii) -x + 1, y + 1/2, -z + 1/2; (iii) -x + 1, y - 1/2, -z + 1/2; (iv) x + 1, -y + 1/2, z + 1/2; (v) -x + 1, -y, -z + 1; (vii) x + 1, y, z; (viii) -x + 1, -y, -z].

**Fig. 2.**
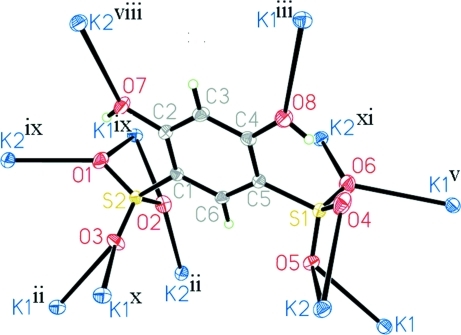
View of chelating/bridging to K+ ions by the sulfonic and phenolic hydroxy groups in the L2-anion, displacement ellipsoids are drawn at 30% probability level. [Symmetry codes: (ii) -x + 1, y + 1/2, -z + 1/2; (iii) -x + 1, y - 1/2, -z + 1/2; (v) -x + 1, -y, -z + 1; (viii) -x + 1, -y, -z (ix) x - 1, -y + 1/2, z - 1/2; (x) x, -y + 1/2, z - 1/2; (xi) x - 1, y, z].

**Fig. 3.**
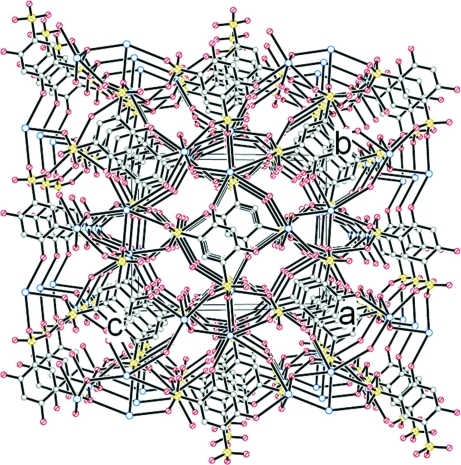
Perspective view of crystal packing along the a axis. Hydrogen atoms are omitted for clarity.

## supplementary crystallographic information

### Comment

The title compound, (I), consists of a three dimensional framework of K^+^ ions coordinated to 4, 6-dihydroxybenzene-1, 3-disulfonate anions. The K^+^ ion is typically seven-coordinated (Deacon *et al.*, 1999), in which the coordination environment around the K^+^ center in (I) is a distorted pentangular dipyramid. As shown in Fig.1, the K1 ion is coordinated by seven O atoms from six 4, 6-dihydroxybenzene-1, 3-disulfonate anions, in which the K—O bond distances range from 2.7160 (14) to 3.0206 (16) Å, with an average bond distance of 2.8460 Å, and the K2 ion is also coordinated by five 4, 6-dihydroxybenzene-1, 3-disulfonate anions and one water molecule, in which the K—O bond distances range from 2.6606 (14) to 3.0518 (16) Å, with an average bond distance of 2.791 Å. The K—O bond distances are similar to the values in other potassium complexes (Hatano *et al.*, 2008; Xie *et al.*, 2006; Zhang *et al.*, 2006). The two sulfonate groups of the 4, 6-dihydroxybenzene-1, 3-disulfonate anions in (I) exhibit a chelating-bridging pentadentate or tetradentate coordination. The O1/O2 atoms and O5/O6 atoms bridge two different K^+^ ions, the other O atoms including those of the hydroxide groups chelate to one type of K^+^ ion (Fig. 2) giving a three dimensional structure (Fig. 3).

### Experimental

KOH (0.14 g, 2.5 mmol) and 4, 6-dihydroxybenzene-1, 3-disulfonate acid (0.91 g, 2.5 mmol) were dissolved in 10 ml of distilled water by vigorous stirring. Filtering solution was evaporated slowly at room temperature. Colorless block crystals of the tilted compound suitable for X-ray analysis were collected in 55% yield (based on potassium) after one week.

### Refinement

H atoms bound to C atoms were placed in calculated positions and treated as riding on their parent atoms, with C—H = 0.93Å, and with U_iso_(H) = 1.2U_eq_(C). Water H atoms were initially located in a difference Fourier map, but they were treated as riding on their parent atoms with O—H = 0.85 Å, and with U_iso_(H) = 1.2U_eq_(O). Hydroxy H atoms were placed in calculated positions and treated as riding on their parent atoms, with O—H = 0.82Å, and with U_iso_(H) = 1.5U_eq_(O).

### Crystal data

**Table:**

[K_2_(C_6_H_4_O_8_S_2_)(H_2_O)]	*F*(000) = 736
*M**_r_* = 364.43	*D*_x_ = 1.989 Mg m^−^^3^
Monoclinic, *P*2_1_/*c*	Mo *K*α radiation, λ = 0.71073 Å
Hall symbol: -P 2ybc	Cell parameters from 2637 reflections
*a* = 7.3427 (3) Å	θ = 2.3–27.4°
*b* = 11.5194 (5) Å	µ = 1.16 mm^−^^1^
*c* = 14.6331 (6) Å	*T* = 292 K
β = 100.495 (1)°	Block, colorless
*V* = 1217.01 (9) Å^3^	0.33 × 0.20 × 0.17 mm
*Z* = 4	

### Data collection

**Table:**

Bruker SMART APEXII CCD area-detector diffractometer	2365 independent reflections
Radiation source: fine-focus sealed tube	2164 reflections with *I* > 2σ(*I*)
graphite	*R*_int_ = 0.013
φ and ω scans	θ_max_ = 26.0°, θ_min_ = 2.3°
Absorption correction: multi-scan (*SADABS*; Sheldrick, 1996)	*h* = −9→8
*T*_min_ = 0.753, *T*_max_ = 0.816	*k* = −14→14
6519 measured reflections	*l* = −13→18

### Refinement

**Table:**

Refinement on *F*^2^	Secondary atom site location: difference Fourier map
Least-squares matrix: full	Hydrogen site location: inferred from neighbouring sites
*R*[*F*^2^ > 2σ(*F*^2^)] = 0.024	H-atom parameters constrained
*wR*(*F*^2^) = 0.071	*w* = 1/[σ^2^(*F*_o_^2^) + (0.0436*P*)^2^ + 0.4024*P*] where *P* = (*F*_o_^2^ + 2*F*_c_^2^)/3
*S* = 1.08	(Δ/σ)_max_ < 0.001
2365 reflections	Δρ_max_ = 0.38 e Å^−^^3^
173 parameters	Δρ_min_ = −0.33 e Å^−^^3^
0 restraints	Extinction correction: *SHELXL97* (Sheldrick, 2008), Fc^*^=kFc[1+0.001xFc^2^λ^3^/sin(2θ)]^-1/4^
Primary atom site location: structure-invariant direct methods	Extinction coefficient: 0.0022 (7)

### Special details

**Table:**

Geometry. All e.s.d.'s (except the e.s.d. in the dihedral angle between two l.s. planes) are estimated using the full covariance matrix. The cell e.s.d.'s are taken into account individually in the estimation of e.s.d.'s in distances, angles and torsion angles; correlations between e.s.d.'s in cell parameters are only used when they are defined by crystal symmetry. An approximate (isotropic) treatment of cell e.s.d.'s is used for estimating e.s.d.'s involving l.s. planes.
Refinement. Refinement of *F*^2^ against ALL reflections. The weighted *R*-factor *wR* and goodness of fit *S* are based on *F*^2^, conventional *R*-factors *R* are based on *F*, with *F* set to zero for negative *F*^2^. The threshold expression of *F*^2^ > σ(*F*^2^) is used only for calculating *R*-factors(gt) *etc*. and is not relevant to the choice of reflections for refinement. *R*-factors based on *F*^2^ are statistically about twice as large as those based on *F*, and *R*- factors based on ALL data will be even larger.

### Fractional atomic coordinates and isotropic or equivalent isotropic displacement parameters (Å^2^)

**Table:**

	*x*	*y*	*z*	*U*_iso_*/*U*_eq_	
K1	0.65409 (5)	0.13314 (3)	0.49915 (3)	0.03113 (13)	
K2	0.92404 (6)	0.02144 (4)	0.28049 (3)	0.03374 (13)	
S1	0.44515 (6)	0.00084 (4)	0.26286 (3)	0.02489 (13)	
S2	0.13059 (5)	0.32694 (3)	0.01439 (3)	0.02338 (13)	
O1	0.0057 (2)	0.32564 (11)	−0.07575 (9)	0.0390 (3)	
O2	0.03127 (18)	0.35943 (11)	0.08772 (10)	0.0349 (3)	
O3	0.29405 (18)	0.39641 (11)	0.01473 (10)	0.0372 (3)	
O4	0.5722 (2)	−0.09468 (13)	0.25379 (10)	0.0431 (4)	
O5	0.5404 (2)	0.10174 (12)	0.30606 (9)	0.0401 (3)	
O6	0.29385 (19)	−0.03620 (13)	0.30678 (9)	0.0410 (3)	
O7	0.0959 (2)	0.11766 (12)	−0.12326 (9)	0.0381 (3)	
H7A	0.0522	0.1834	−0.1271	0.046*	
O8	0.3915 (2)	−0.15510 (11)	0.08903 (10)	0.0383 (3)	
H8A	0.4576	−0.1604	0.1404	0.046*	
O9	0.8087 (4)	0.21939 (16)	0.19154 (15)	0.0948 (8)	
H9A	0.7450	0.2678	0.2163	0.114*	
H9B	0.8358	0.2336	0.1386	0.114*	
C1	0.2023 (2)	0.18131 (14)	0.03585 (11)	0.0225 (3)	
C2	0.1799 (2)	0.09683 (15)	−0.03415 (11)	0.0254 (4)	
C3	0.2454 (2)	−0.01451 (15)	−0.01379 (12)	0.0292 (4)	
H3A	0.2321	−0.0700	−0.0607	0.035*	
C4	0.3303 (2)	−0.04435 (14)	0.07530 (12)	0.0258 (3)	
C5	0.3490 (2)	0.03891 (14)	0.14670 (11)	0.0233 (3)	
C6	0.2866 (2)	0.15100 (14)	0.12554 (12)	0.0241 (3)	
H6A	0.3015	0.2069	0.1722	0.029*	

### Atomic displacement parameters (Å^2^)

**Table:**

	*U*^11^	*U*^22^	*U*^33^	*U*^12^	*U*^13^	*U*^23^
K1	0.0284 (2)	0.0278 (2)	0.0368 (2)	−0.00127 (15)	0.00475 (16)	0.00329 (15)
K2	0.0373 (2)	0.0326 (2)	0.0297 (2)	0.00037 (16)	0.00155 (17)	−0.00276 (16)
S1	0.0255 (2)	0.0254 (2)	0.0225 (2)	0.00017 (16)	0.00132 (16)	0.00241 (15)
S2	0.0237 (2)	0.0199 (2)	0.0257 (2)	0.00147 (15)	0.00221 (16)	0.00099 (15)
O1	0.0470 (8)	0.0309 (7)	0.0329 (7)	0.0074 (6)	−0.0093 (6)	0.0026 (6)
O2	0.0364 (7)	0.0305 (7)	0.0402 (8)	0.0088 (5)	0.0131 (6)	−0.0009 (5)
O3	0.0319 (7)	0.0263 (7)	0.0538 (9)	−0.0039 (5)	0.0092 (6)	0.0049 (6)
O4	0.0458 (8)	0.0437 (8)	0.0363 (7)	0.0193 (7)	−0.0020 (6)	0.0016 (6)
O5	0.0492 (8)	0.0366 (7)	0.0291 (7)	−0.0113 (6)	−0.0071 (6)	0.0010 (6)
O6	0.0364 (7)	0.0531 (9)	0.0349 (7)	−0.0057 (6)	0.0100 (6)	0.0096 (6)
O7	0.0549 (9)	0.0336 (7)	0.0219 (6)	0.0099 (6)	−0.0029 (6)	−0.0033 (5)
O8	0.0521 (8)	0.0218 (6)	0.0366 (7)	0.0081 (6)	−0.0033 (6)	−0.0020 (5)
O9	0.178 (3)	0.0389 (10)	0.0766 (14)	0.0139 (13)	0.0474 (15)	0.0119 (9)
C1	0.0222 (8)	0.0205 (8)	0.0249 (8)	0.0014 (6)	0.0041 (6)	0.0008 (6)
C2	0.0259 (8)	0.0279 (9)	0.0220 (8)	0.0002 (7)	0.0029 (6)	−0.0012 (7)
C3	0.0343 (10)	0.0250 (9)	0.0269 (9)	0.0011 (7)	0.0023 (7)	−0.0073 (7)
C4	0.0270 (8)	0.0202 (8)	0.0300 (9)	0.0001 (7)	0.0046 (7)	−0.0015 (7)
C5	0.0233 (8)	0.0236 (8)	0.0223 (8)	0.0002 (6)	0.0019 (6)	0.0007 (6)
C6	0.0256 (8)	0.0224 (8)	0.0234 (8)	−0.0004 (6)	0.0019 (6)	−0.0036 (6)

### Geometric parameters (Å, °)

**Table:**

K1—O3^i^	2.7160 (14)	O1—K2^viii^	2.7256 (13)
K1—O8^ii^	2.7514 (13)	O1—K1^viii^	3.0207 (16)
K1—O3^iii^	2.7660 (14)	O2—K2^ii^	2.6607 (14)
K1—O5	2.8200 (14)	O2—K1^viii^	2.8372 (14)
K1—O2^iv^	2.8372 (14)	O3—K1^ix^	2.7160 (14)
K1—O6^v^	3.0104 (14)	O3—K1^ii^	2.7660 (14)
K1—O1^iv^	3.0207 (16)	O6—K2^x^	2.7535 (15)
K1—O9^i^	3.307 (2)	O6—K1^v^	3.0104 (14)
K1—S2^iv^	3.4954 (6)	O7—C2	1.359 (2)
K2—O2^iii^	2.6607 (14)	O7—K2^vii^	2.7856 (13)
K2—O9	2.6851 (19)	O7—H7A	0.8204
K2—O1^iv^	2.7256 (13)	O8—C4	1.356 (2)
K2—O6^vi^	2.7535 (15)	O8—K1^iii^	2.7514 (13)
K2—O7^vii^	2.7856 (13)	O8—H8A	0.8194
K2—O4	2.8720 (16)	O9—K1^ix^	3.307 (2)
K2—O5	3.0521 (16)	O9—H9A	0.8500
K2—S1	3.4867 (6)	O9—H9B	0.8502
S1—O5	1.4417 (13)	C1—C6	1.390 (2)
S1—O6	1.4455 (14)	C1—C2	1.401 (2)
S1—O4	1.4641 (14)	C2—C3	1.383 (2)
S1—C5	1.7729 (16)	C3—C4	1.382 (2)
S2—O3	1.4418 (13)	C3—H3A	0.9300
S2—O2	1.4519 (13)	C4—C5	1.406 (2)
S2—O1	1.4628 (13)	C5—C6	1.386 (2)
S2—C1	1.7691 (16)	C6—H6A	0.9300
S2—K1^viii^	3.4954 (6)		
			
O3^i^—K1—O8^ii^	96.50 (4)	O1^iv^—K2—S1	100.27 (3)
O3^i^—K1—O3^iii^	91.82 (4)	O6^vi^—K2—S1	161.72 (4)
O8^ii^—K1—O3^iii^	147.81 (5)	O7^vii^—K2—S1	89.95 (3)
O3^i^—K1—O5	87.31 (5)	O4—K2—S1	24.24 (3)
O8^ii^—K1—O5	69.84 (4)	O5—K2—S1	24.33 (3)
O3^iii^—K1—O5	79.60 (4)	O2^iii^—K2—K1	71.35 (3)
O3^i^—K1—O2^iv^	148.16 (4)	O9—K2—K1	87.63 (5)
O8^ii^—K1—O2^iv^	102.29 (4)	O1^iv^—K2—K1	44.92 (3)
O3^iii^—K1—O2^iv^	85.83 (4)	O6^vi^—K2—K1	123.37 (3)
O5—K1—O2^iv^	123.22 (4)	O7^vii^—K2—K1	146.39 (3)
O3^i^—K1—O6^v^	80.36 (4)	O4—K2—K1	73.30 (3)
O8^ii^—K1—O6^v^	139.02 (4)	O5—K2—K1	41.41 (3)
O3^iii^—K1—O6^v^	73.03 (4)	S1—K2—K1	58.329 (11)
O5—K1—O6^v^	149.45 (4)	O2^iii^—K2—K1^xi^	37.90 (3)
O2^iv^—K1—O6^v^	68.57 (4)	O9—K2—K1^xi^	144.42 (6)
O3^i^—K1—O1^iv^	163.66 (4)	O1^iv^—K2—K1^xi^	70.77 (3)
O8^ii^—K1—O1^iv^	74.31 (4)	O6^vi^—K2—K1^xi^	42.14 (3)
O3^iii^—K1—O1^iv^	89.44 (4)	O7^vii^—K2—K1^xi^	107.18 (3)
O5—K1—O1^iv^	76.90 (4)	O4—K2—K1^xi^	113.91 (3)
O2^iv^—K1—O1^iv^	48.18 (4)	O5—K2—K1^xi^	124.94 (3)
O6^v^—K1—O1^iv^	115.50 (4)	S1—K2—K1^xi^	126.161 (14)
O3^i^—K1—O9^i^	100.44 (5)	K1—K2—K1^xi^	86.205 (11)
O8^ii^—K1—O9^i^	86.41 (5)	O5—S1—O6	113.19 (9)
O3^iii^—K1—O9^i^	122.58 (5)	O5—S1—O4	112.30 (9)
O5—K1—O9^i^	155.78 (5)	O6—S1—O4	111.87 (9)
O2^iv^—K1—O9^i^	55.98 (6)	O5—S1—C5	107.31 (8)
O6^v^—K1—O9^i^	54.73 (5)	O6—S1—C5	107.16 (8)
O1^iv^—K1—O9^i^	92.56 (5)	O4—S1—C5	104.37 (8)
O3^i^—K1—S2^iv^	171.66 (4)	O5—S1—K2	60.70 (6)
O8^ii^—K1—S2^iv^	87.03 (3)	O6—S1—K2	146.47 (6)
O3^iii^—K1—S2^iv^	89.13 (3)	O4—S1—K2	53.65 (6)
O5—K1—S2^iv^	101.01 (3)	C5—S1—K2	105.92 (6)
O2^iv^—K1—S2^iv^	23.71 (3)	O3—S2—O2	112.60 (8)
O6^v^—K1—S2^iv^	92.00 (3)	O3—S2—O1	113.16 (9)
O1^iv^—K1—S2^iv^	24.59 (2)	O2—S2—O1	110.63 (8)
O9^i^—K1—S2^iv^	72.17 (5)	O3—S2—C1	107.76 (8)
O3^i^—K1—K1^v^	46.45 (3)	O2—S2—C1	106.66 (8)
O8^ii^—K1—K1^v^	133.97 (3)	O1—S2—C1	105.53 (8)
O3^iii^—K1—K1^v^	45.37 (3)	O3—S2—K1^viii^	138.60 (6)
O5—K1—K1^v^	80.54 (3)	O2—S2—K1^viii^	51.80 (6)
O2^iv^—K1—K1^v^	123.52 (3)	O1—S2—K1^viii^	59.22 (6)
O6^v^—K1—K1^v^	70.68 (3)	C1—S2—K1^viii^	113.46 (6)
O1^iv^—K1—K1^v^	132.54 (3)	S2—O1—K2^viii^	134.67 (8)
O9^i^—K1—K1^v^	121.30 (4)	S2—O1—K1^viii^	96.20 (7)
S2^iv^—K1—K1^v^	133.906 (18)	K2^viii^—O1—K1^viii^	95.50 (4)
O3^i^—K1—K2	128.22 (3)	S2—O2—K2^ii^	134.76 (8)
O8^ii^—K1—K2	87.08 (3)	S2—O2—K1^viii^	104.48 (7)
O3^iii^—K1—K2	63.75 (3)	K2^ii^—O2—K1^viii^	106.93 (4)
O5—K1—K2	45.71 (3)	S2—O3—K1^ix^	138.81 (8)
O2^iv^—K1—K2	78.64 (3)	S2—O3—K1^ii^	132.27 (8)
O6^v^—K1—K2	126.89 (3)	K1^ix^—O3—K1^ii^	88.18 (4)
O1^iv^—K1—K2	39.58 (2)	S1—O4—K2	102.11 (8)
O9^i^—K1—K2	131.33 (5)	S1—O5—K1	125.04 (8)
S2^iv^—K1—K2	59.350 (11)	S1—O5—K2	94.98 (7)
K1^v^—K1—K2	96.882 (15)	K1—O5—K2	92.88 (4)
O3^i^—K1—K2^xi^	116.87 (3)	S1—O6—K2^x^	133.23 (8)
O8^ii^—K1—K2^xi^	135.14 (3)	S1—O6—K1^v^	123.58 (7)
O3^iii^—K1—K2^xi^	64.45 (3)	K2^x^—O6—K1^v^	100.01 (4)
O5—K1—K2^xi^	136.03 (3)	C2—O7—K2^vii^	129.60 (11)
O2^iv^—K1—K2^xi^	35.17 (3)	C2—O7—H7A	109.4
O6^v^—K1—K2^xi^	37.85 (3)	K2^vii^—O7—H7A	120.9
O1^iv^—K1—K2^xi^	78.24 (3)	C4—O8—K1^iii^	139.13 (11)
O9^i^—K1—K2^xi^	59.97 (4)	C4—O8—H8A	109.4
S2^iv^—K1—K2^xi^	56.355 (10)	K1^iii^—O8—H8A	111.4
K1^v^—K1—K2^xi^	90.518 (14)	K2—O9—K1^ix^	151.40 (8)
K2—K1—K2^xi^	93.795 (11)	K2—O9—H9A	120.0
O2^iii^—K2—O9	158.97 (6)	K1^ix^—O9—H9A	84.0
O2^iii^—K2—O1^iv^	85.02 (4)	K2—O9—H9B	120.0
O9—K2—O1^iv^	80.27 (6)	K1^ix^—O9—H9B	39.3
O2^iii^—K2—O6^vi^	75.01 (4)	H9A—O9—H9B	120.0
O9—K2—O6^vi^	119.25 (7)	C6—C1—C2	119.26 (15)
O1^iv^—K2—O6^vi^	88.58 (5)	C6—C1—S2	118.21 (12)
O2^iii^—K2—O7^vii^	100.15 (4)	C2—C1—S2	122.52 (13)
O9—K2—O7^vii^	97.10 (5)	O7—C2—C3	116.73 (15)
O1^iv^—K2—O7^vii^	168.61 (5)	O7—C2—C1	123.46 (15)
O6^vi^—K2—O7^vii^	83.04 (4)	C3—C2—C1	119.80 (15)
O2^iii^—K2—O4	76.18 (4)	C4—C3—C2	120.87 (16)
O9—K2—O4	97.64 (7)	C4—C3—H3A	119.6
O1^iv^—K2—O4	118.17 (5)	C2—C3—H3A	119.6
O6^vi^—K2—O4	138.28 (4)	O8—C4—C3	117.02 (15)
O7^vii^—K2—O4	73.09 (4)	O8—C4—C5	123.15 (15)
O2^iii^—K2—O5	96.80 (4)	C3—C4—C5	119.83 (15)
O9—K2—O5	65.50 (6)	C6—C5—C4	119.06 (15)
O1^iv^—K2—O5	77.75 (4)	C6—C5—S1	120.16 (13)
O6^vi^—K2—O5	164.73 (4)	C4—C5—S1	120.76 (13)
O7^vii^—K2—O5	111.39 (4)	C5—C6—C1	121.14 (15)
O4—K2—O5	47.96 (4)	C5—C6—H6A	119.4
O2^iii^—K2—S1	89.74 (3)	C1—C6—H6A	119.4
O9—K2—S1	78.29 (6)		

Symmetry codes: (i) *x*, −*y*+1/2, *z*+1/2; (ii) −*x*+1, *y*+1/2, −*z*+1/2; (iii) −*x*+1, *y*−1/2, −*z*+1/2; (iv) *x*+1, −*y*+1/2, *z*+1/2; (v) −*x*+1, −*y*, −*z*+1; (vi) *x*+1, *y*, *z*; (vii) −*x*+1, −*y*, −*z*; (viii) *x*−1, −*y*+1/2, *z*−1/2; (ix) *x*, −*y*+1/2, *z*−1/2; (x) *x*−1, *y*, *z*; (xi) −*x*+2, −*y*, −*z*+1.

### Hydrogen-bond geometry (Å, °)

**Table:**

*D*—H···*A*	*D*—H	H···*A*	*D*···*A*	*D*—H···*A*
O8—H8A···O4	0.82	1.88	2.625 (2)	151
O7—H7A···O1	0.82	1.86	2.613 (2)	152
O9—H9A···O6^ii^	0.85	2.29	2.916 (2)	130
O9—H9B···O2^vi^	0.85	2.26	2.913 (3)	134

Symmetry codes:  (ii) −*x*+1, *y*+1/2, −*z*+1/2; (vi) *x*+1, *y*, *z*.
